# Strength of Hydrogen Bond Network Takes Crucial Roles in the Dissociation Process of Inhibitors from the HIV-1 Protease Binding Pocket

**DOI:** 10.1371/journal.pone.0019268

**Published:** 2011-04-29

**Authors:** Dechang Li, Baohua Ji, Keh-Chih Hwang, Yonggang Huang

**Affiliations:** 1 School of Aerospace, Department of Engineering Mechanics, Tsinghua University, Beijing, China; 2 Biomechanics and Biomaterials Laboratory, Department of Applied Mechanics, Beijing Institute of Technology, Beijing, China; 3 Department of Civil and Environmental Engineering, Northwestern University, Evanston, Illinois, United States of America; Massachusetts Institute of Technology, United States of America

## Abstract

To understand the underlying mechanisms of significant differences in dissociation rate constant among different inhibitors for HIV-1 protease, we performed steered molecular dynamics (SMD) simulations to analyze the entire dissociation processes of inhibitors from the binding pocket of protease at atomistic details. We found that the strength of hydrogen bond network between inhibitor and the protease takes crucial roles in the dissociation process. We showed that the hydrogen bond network in the cyclic urea inhibitors AHA001/XK263 is less stable than that of the approved inhibitor ABT538 because of their large differences in the structures of the networks. In the cyclic urea inhibitor bound complex, the hydrogen bonds often distribute at the flap tips and the active site. In contrast, there are additional accessorial hydrogen bonds formed at the lateral sides of the flaps and the active site in the ABT538 bound complex, which take crucial roles in stabilizing the hydrogen bond network. In addition, the water molecule W301 also plays important roles in stabilizing the hydrogen bond network through its flexible movement by acting as a collision buffer and helping the rebinding of hydrogen bonds at the flap tips. Because of its high stability, the hydrogen bond network of ABT538 complex can work together with the hydrophobic clusters to resist the dissociation, resulting in much lower dissociation rate constant than those of cyclic urea inhibitor complexes. This study may provide useful guidelines for design of novel potent inhibitors with optimized interactions.

## Introduction

Human immunodeficiency virus type 1 (HIV-1) protease is a symmetric homo-dimeric aspartyl protease, which cleaves the *gag* and *pol* viral polyproteins at its active site to process viral maturation [1]. Due to its indispensability for the infection of the virus, the HIV-1 protease (HIV-1 PR) is one of the primary targets of anti-AIDS therapy [2]. However, new potent inhibitors are still often needed because of the selection of inhibitor-resistant variants of the protease (PR), which leads to limited long term use of current inhibitors. To improve the efficacy of inhibitors, many efforts had been paid for studying the kinetic processes of association and dissociation of the interaction between inhibitors and the HIV-1 PR. It was found that current inhibitors, including the approved and the non-approved, exhibit distinct kinetic processes, of which the underlying mechanisms are of primary importance for structure-based drug design. For instance, experimental results indicated that there are a wide range of association rate and dissociation rate constants in different inhibitors, e.g., 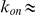
10^9^∼10^10^ M^−1^s^−1^ and 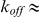
∼100 s^−1^ for cyclic urea inhibitors, while 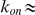
10^5^∼10^6^ M^−1^s^−1^ and 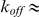
10^−3^∼10^−4^ s^−1^ for the approved inhibitors [3]. To understand these significant differences in the association rate and dissociation rate constants has been a primary impetus behind intensive studies.

The effectiveness of inhibitors is often denoted by the compound parameter, 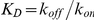 [3], which suggests that the efficacy optimization of new potent inhibitors should be guided by aiming for high association and low dissociation rates simultaneously rather than high association rate alone [3]. Molecular dynamics (MD) simulation, as a powerful tool for studying the kinetic process of inhibitors, can be used for identifying crucial factors that influence the association and dissociation processes of inhibitors during the structure-based drug design. To understand the binding behaviors of inhibitors with the PR, both full-atom and coarse grained (CG) MD methods were adopted to simulate the dynamics of free PR and PR-inhibitor complex [4], [5], [6], [7], [8], [9], [10], [11]. Chang et al. [12] studied the binding pathway of a cyclic urea inhibitor XK263 and a substrate using CG MD simulations. Pietrucci et al. [13] studied the binding mechanism of a substrate using MD simulations with a so-called bias-exchange metadynamics technique. Li et al. [8] and Cheng et al. [14] further simulated the binding process of various inhibitors of different binding energy, molecular size and rigidity with CG MD simulations. They showed that the binding process was gated by the opening dynamics of the flaps of the PR, and this gated binding processes can be significantly affected by molecular properties of inhibitors, such as inhibitors' size, topology and stiffness. These studies to some extent explained the mechanisms for the wide variety of association rate constants in different inhibitors.

Compared with the association process, the dissociation process of inhibitors is much less understood. The experiments by Maschera et al. [15] indicated that the mutations of the protease often decreased the effectiveness of inhibitors by significantly increasing the dissociation rate constants, but tinily influencing the association rate constants. This result indicates that the dissociation rate is more sensitive to the mutations, in which the underlying mechanisms are important for potent inhibitor design. In addition, Markgren et al. [3] showed that the affinities of the cyclic urea inhibitors were often limited by its ultra fast dissociation rates. To study the dissociation processes, Trylska et al. [16] studied the dynamics of product release process with CG MD simulations. Sadiq et al. [17] simulated the early stages of release process of inhibitors by all-atom MD simulations and found that there is a lateral escaping tendency of inhibitors assisted by mutations of the PR. Li et al. [10] studied the role of the sub-nanosecond local dynamics of flap tips in the stability of the bound complexes and showed that the local dynamics are affected by broken and formation of hydrogen bonds between flap tips and inhibitors. They found that the water molecule W301 within the binding pocket of bound complex plays crucial roles in the binding stability of inhibitors. To the best of our knowledge, there is no report on studies of entire dissociation processes of inhibitors from the HIV-1 PR with full atom simulations. Compared to the coarse grained simulation, the full-atom simulation allows us to track the atomistic details in the dynamics of system. The information in atomistic details of the dissociation processes of inhibitors from HIV-1 PR is of importance for optimizing the interactions during inhibitor design.

In this paper, we will apply the steered molecular dynamics (SMD) simulations to study the dissociation behaviors of inhibitors under external force considering that the time scale of dissociation processes is beyond the limit of classical MD simulations. For example, the timescale of the natural dissociation process of cyclic urea inhibitors is estimated to be ∼10 ms considering their dissociation rate constants being ∼100 s^−1^ [3], while the timescale the classical full-atom MD simulations can achieve is typically nano- to micro-seconds. By applying a force to the system, the dissociation processes can be largely accelerated in the SMD simulation. This kind of single-molecule pulling simulations or experiments has been widely used to investigate the ligand-receptor interactions [18], [19], the protein-protein interactions [20], as well as the unfolding processes of proteins [21], [22]. Particularly, they can be used to calculate the profile of the free energy landscape of the molecular interaction [23], [24], [25], [26], [27], [28], [29], [30]. Here we will apply the SMD simulation to study the critical interactions between inhibitors and the PR at atomistic details. The umbrella sampling method [31] will be applied to calculate the profiles of the energy landscapes of the systems. The aim of this work is to find the underlying mechanisms at atomistic details that influence the dissociation rate of different inhibitors, through which to obtain useful guidelines for design of novel potent inhibitors.

## Methods

### MD simulations

The structures for the inhibitor bound complexes were retrieved from Protein Data Bank with PDB codes: 1AJX [32] for AHA001 bound complex, 1HVR [33] for XK263 bound complex and 1HXW [34] for ABT538 bound complex. The catalytic Asp side chains of the bound complex were protonated according to the experiments and theoretical calculations, i.e. both side chains of Asp25/Asp25′ were protonated for AHA001 and XK263 bound complex [35], and only one of the Asp25/Asp25′ was protonated for ABT538 bound complex [36].

The MD simulations were performed using Gromacs package [37] with the AMBER force field of ffamber99 [38], in which the all-atom force field parameters of inhibitors were obtained by the ANTECHAMBER module and GAFF [39] with AM1-BCC [40] charges in AMBER package [41].

Each system was solvated in a 90×80×80 Å^3^ water box, with about 15,000 water molecules. Appropriate chlorine ions were added to neutralize the system. Particle Mesh Ewald (PME) method [42] was used to calculate the long-range electrostatic interactions. The systems were minimized firstly using steepest descent algorithm by 10,000 steps. Then, the system was gradually heated from 200K to 300K in 200 ps, while positional restraints were used for the heavy atoms of the protease and the inhibitor. The restraint force constants were gradually decreased from 1660 pN/nm to 0 pN/nm in a few stages. All production simulations were conducted at 300K and 1 bar with the Berendsen algorithm. The LINCS algorithm [43] was applied to constrain the covalent bonds with H-atoms. The time step of the simulations is 2.0 fs. The cut-off of the non-bonded interactions was set to be 10 Å. The non-bonded pairs were updated in every 10 steps.

### Steered MD simulations

Structures from the result of each 40 ns MD simulations were used as starting configurations for steered MD (SMD) simulations. We used a constant pulling speed to apply force to the system. The steered “dummy” atom was attached via a spring to the center of mass (COM) of the inhibitors and moves at a constant velocity. In the SMD simulation, the applied force is given by 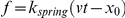, where 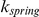 is the spring constant, 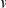 is the pulling velocity and *t* is the simulation time. Thus the force rate can be obtained as 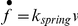. In order to study the binding strength of the bound complexes, we carried out a series of computational experiments by using a large range of force rate over six orders of magnitude, by systematically varying the spring constant (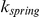 = 6947.7 pN/nm, 3473.9 pN/nm, 694.8 pN/nm, and 347.4 pN/nm) and the pulling velocity (from 200 nm/ns to 0.02 nm/ns), which is from 1.4×10^6^ pN/ns to 6.95 pN/ns. Each force rate was simulated more than twice to calculate the average rupture forces and the deviations (see Table S1).

**Figure 1:**
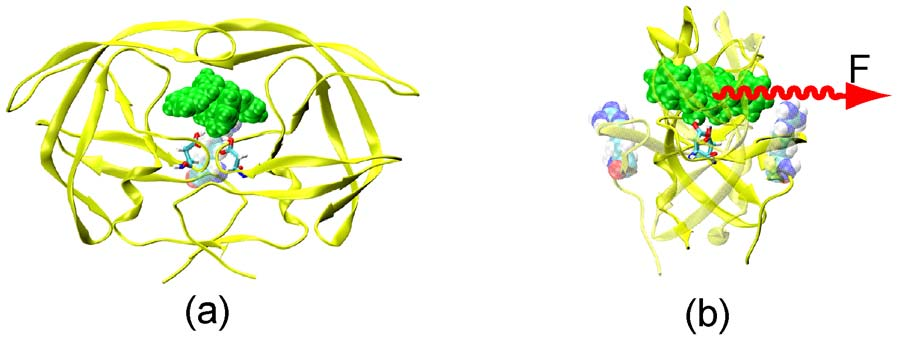
Carton draws of the HIV-1 protease in complex with inhibitor. Residue Arg8 and Arg8′ were represented by VDW spheres. The inhibitor was represented by green VDW spheres. The red arrow shows the directions of the external forces implemented in the SMD simulations. (a) The front view of the bound complex; (b) The side view of the bound complex with an external force applied on the inhibitor.

Because Sadiq et. al. [17] showed that the inhibitor tended to laterally escape from the binding pocket, and Trylska et al. [16] also showed that the peptide product would laterally slide out from the binding pocket, we chose the pulling direction along the lateral direction, depicted by the vector from the COM of residue Arg8 to the COM of residue Arg8′ (see Figure 1).

To prevent translational and rotational displacement of the PR molecule, several C_α_ atoms of the PR were held by positional restraints, including C_α_ atoms of N- and C-termini residues Pro1, Phe99, Pro1′ and Phe99′, as well as Arg8, Leu23, Pro81 and Asp30′ at the back end of the PR.

### Umbrella sampling

From the SMD simulation trajectories, snapshots were taken to generate the starting configurations for the umbrella sampling windows. For each inhibitor bound complex system, the simulation trajectory with 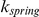 = 694.8 pN/nm and pulling velocity 0.02 nm/ns was chosen to apply the umbrella sampling analysis. An asymmetrical distribution of sampling windows was used, such that the window spacing was about 0.5 Å when the COM separation between the inhibitor and the PR active site (i.e. residues Asp25 and Asp25′) below 10 Å, while that was about 1 Å when the COM separation beyond 10 Å. Such spacing allowed for sampling in detail at smaller COM distance, which resulted in about 40 windows. In each window, 5 ns of MD simulation was performed so that a total simulation time of ∼200 ns was utilized for umbrella sampling in each system. Analysis of results was performed with the weighted histogram analysis method (WHAM) [44].

### Hydrogen Bond Criteria

To determine whether a hydrogen bond (H-bond) exists between donor and acceptor, a geometrical criterion was adopted, in which the formation of a hydrogen bond was defined by both atom distance and bond orientation. For instance, the combination of donor D, hydrogen H, and acceptor A with a D-H· · ·A configuration was regarded as a hydrogen bond when the distance between donor D and acceptor A was shorter than 3.5 Å as well as the bond angle H-D· · ·A was smaller than 60.0°.

## Results

### H-bonds and hydrophobic interactions between the PR and inhibitors

**Figure 2:**
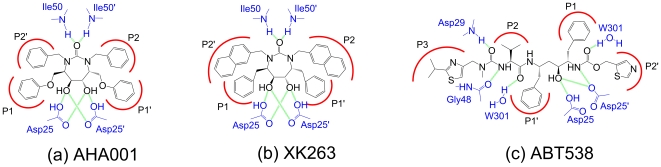
Illustration of molecular structures of inhibitors and their H-bonds and hydrophobic interactions with the protease. (a) AHA001, (b) XK263 and (c) ABT538. Sidechains of the inhibitors are labeled as P1, P2, etc., and these sidechains can insert into the sub-sites of the protease (labeled as S1, S2, etc.) to form hydrophobic clusters. The residues in protease are labeled in blue in (a), (b), and (c). The possible H-bonds between the protease and inhibitors are labeled in green.

Figure 2 (a), (b) and (c) illustrated the H-bonds and hydrophobic interactions between the inhibitors and the PR for three complexes, AHA001-PR, XK263-PR and ABT538-PR, respectively. Table 1 shows the root mean square deviation (RMSD) values of protease C_α_ atoms of these complexes after 40 ns MD simulations without applying external forces. The low RMSD values indicated that the structures of the complexes were stable and the configurations were close to the initial structures. The time average number and the spatial distributions of H-bonds between the protease and inhibitors were given in Table 1 (also see Figure 2 (a), (b) and (c)). For the cyclic urea inhibitors (i.e. AHA001 and XK263), they formed one H-bond on average with the flap tips (denoted by Ile50/Ile50′:N–AHA001:O for AHA001, see Figure 2 (a); and Ile50/Ile50′:N–XK263:O for XK263, see Figure 2 (b)), while the inhibitor ABT538 formed about two H-bonds with flap tips through a so-called W301 water molecule (denoted by Ile50/Ile50′:N–W301–ABT538, see Figure 2 (c)). In addition, two more H-bonds were formed between ABT538 and residues Asp29 and Gly48 at the lateral sides of the protease (denoted by Asp29:N–ABT538:O42 and Gly48:O–ABT538:N16, respectively). We note that all these three inhibitors formed multiple H-bonds with residues Asp25 and Asp25′ at the active site (denoted by Asp25/Asp25′–ABT538, Asp25/Asp25′–AHA001 and Asp25/Asp25′–XK263, respectively, see Figure 2 (a) to (c)).

**Table 1:** RMSD values of the protease C_α_ atoms and number of H-bonds between the protease and the inhibitors after 40 ns MD simulations.

Complex	RMSD (Å)	Total number of H-bonds	Individual number of H-bonds
AHA001-PR	1.66±0.09	4.54±0.62	Asp25/Asp25′–AHA001: (3.61±0.54)
			Ile50/Ile50′:N–AHA001:O: (0.93±0.33)
XK263-PR	1.37±0.18	4.56±0.87	Asp25/Asp25′–XK263: (3.56±0.65)
			Ile50/Ile50′:N–XK263:O: (1.00±0.50)
ABT538-PR	1.11±0.14	6.38±0.72	Asp25/Asp25′–ABT538: (2.75±0.47)
			Asp29:N–ABT538:O42 : (0.97±0.17)
			Gly48:O–ABT538:N16: (0.91±0.29)
			Ile50/Ile50′:N–W301–ABT538: (1.75±0.44)

Besides H-bond interactions, inhibitors can also have hydrophobic interaction with the PR. There were several hydrophobic clusters formed between the sidechains of the inhibitors and the subsites of the protease, which can be represented by the distances between the hydrophobic groups in the clusters (see Figure 2 (a) to (c)). Through these interactions, the inhibitors bind at the active site so that the protease can be restricted to the closed configuration.

### SMD simulations of the dissociation processes of inhibitors from the PR binding pocket AHA001 and XK263

**Figure 3:**
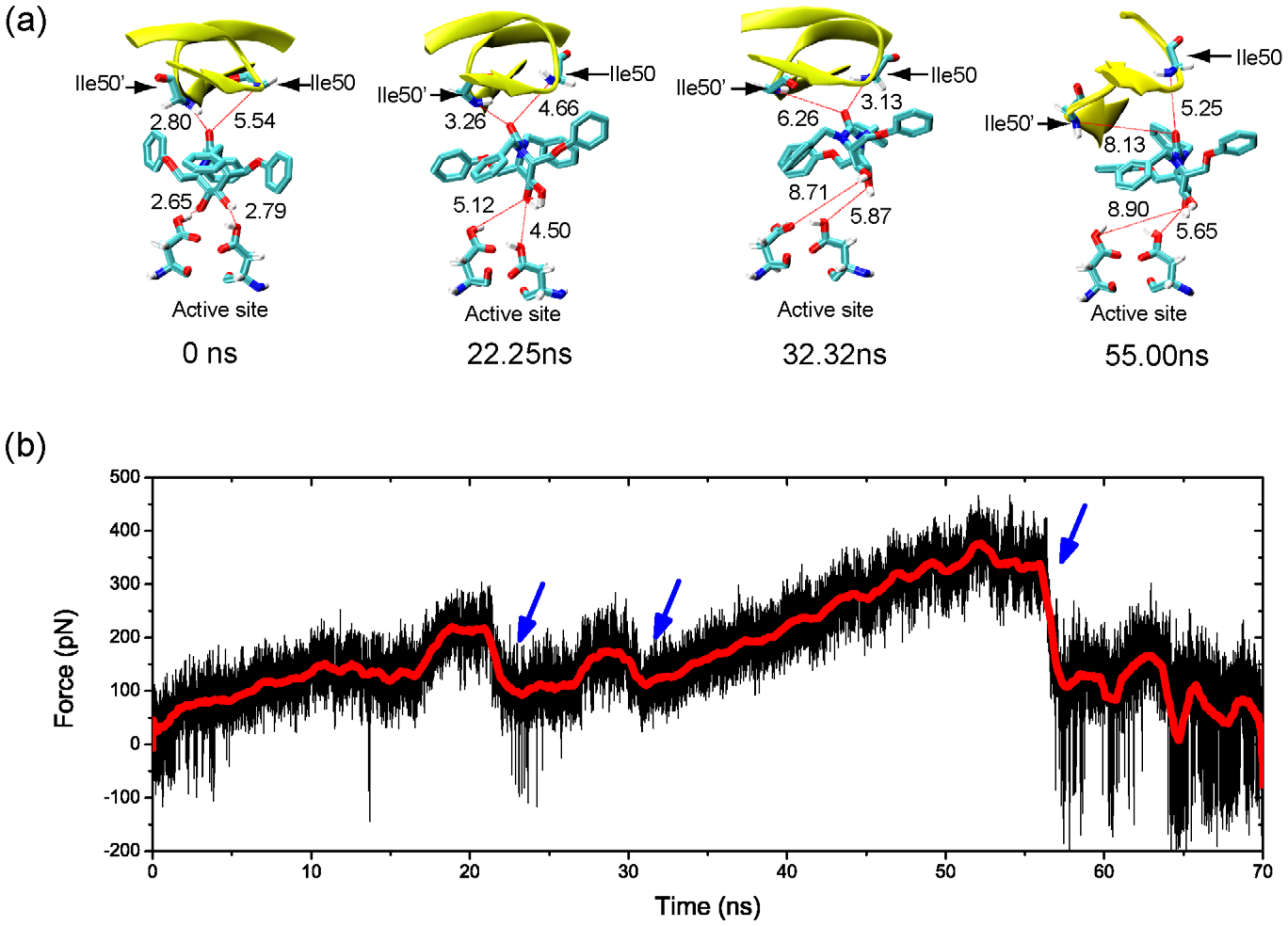
Dissociation process of AHA001 from the protease. (a) Snapshots of AHA001 escaping from the binding pocket of the protease under the external pulling force. The numbers in the panel indicate the lengths of the H-bonds, and the simulation times are given at the bottom of each snapshot. (b) The pulling force of the AHA001 bound complex during the SMD simulation. The red curve is the average values with a running average time window of 500 ps. The blue arrows indicate the critical points of force dropping during the simulation.

**Figure 4:**
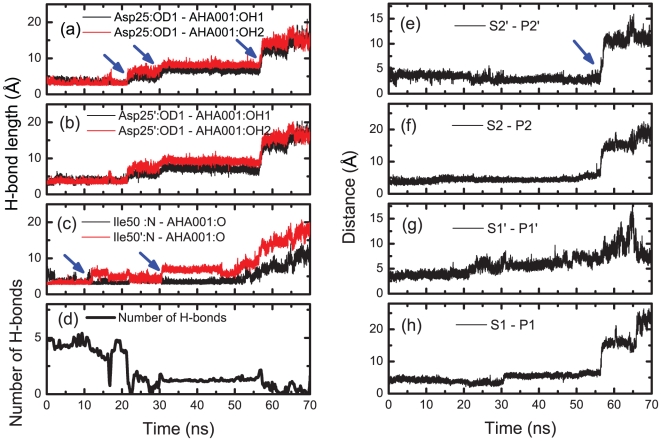
Analyses of the dynamics of H-bond interaction and hydrophobic interactions in AHA001 bound complex. (a) to (c): The H-bond lengths between the protease and the inhibitor AHA001 during the SMD simulation. (d) Evolution of the number of H-bonds between the protease and the inhibitor AHA001 during the simulation. (e) to (h): The distances between the subsites (S) of protease and the sidechains (P) of inhibitor AHA001 during the simulation, which were used to monitor the state of the hydrophobic clusters. The blue arrows indicate the critical points of bond length change during the simulation.

Figure 3 (a) shows the snapshots of AHA001 escaping from the active site under the external forces along the pulling pathway (see Figure 1). When the external force was small at the initial stage, the H-bonds at the flap tips firstly became unstable, see Figures 3 and 4 at 0∼20 ns. The average H-bond number was about 4 (see Figure 4 (d)). With increasing of the pulling force to a critical value, the H-bonds at the active site (Asp25/Asp25′–AHA001) started to rupture, and the average H-bond number decreased to 1, see Figure 3 (a) at 22.25 ns and Figure 4 (a) to (d). It was noted that the three H-bonds of Asp25/Asp25′–AHA001 ruptured simultaneously. With rupture of the H-bond network, the loading force dropped abruptly, and the inhibitor was pulled to deviate from the binding site, see Figure 3 (b) at about 22 ns and 30 ns. Afterwards, the dissociation process of inhibitor was mainly resisted by the hydrophobic interaction between the PR's subsites and AHA001's sidechains via the hydrophobic clusters (i.e., clusters P1-S1, P1′-S1′, P2-S2, P2′-S2′, see Figure 2 (a)). At last, the inhibitor AHA001 was completely pulled out from the binding pocket, and the loading force dropped to zero. Here the hydrophobic interactions were measured by the center-of-mass distance between two hydrophobic groups [17]. We monitored the evolutions of the distance during the simulation (see Figure 4 (e) to (h)). When the distances was larger than 10 Å so that one or more layers of water molecules can enter the space between them and separate them (the size of each hydrophobic group is about 2∼3 Å), the hydrophobic interactions were considered to be ruptured.

The dissociation process of inhibitor XK263 was similar to that of AHA001. Firstly, the H-bonds at the flap tips became less stable under the pulling force. Secondly, the H-bonds between XK263 and the active site (Asp25/Asp25′-XK263) ruptured at a critical value of the pulling force, as shown in Figure S1 (a) at 38.82 ns (also see Figure S2 (a) to (d)), which then caused the failure of the whole H-bond network. Thereafter, the inhibitor slipped away from the active site, which caused a big drop in the loading force at ∼40 ns (see Figure S1 (b)). The pulling force finally dropped to zero after the rupture of the hydrophobic clusters between inhibitors and the protease. There were also some differences between XK263 and AHA001. The force value for the H-bonds' failure at active site (indicated by the first tip value of the force-time curve) of XK263 is higher than that of AHA001, according to the comparison between Figure 3 (b) and Figure S1 (b). In addition, the rupture of the H-bonds at the active site in XK263 bound complex occurred later than that in AHA001 bound complex. For AHA001 complex, most of the hydrophobic clusters ruptured after the failure of the H-bonds at active site (see Figure 4). However, for XK263 complex, most of the hydrophobic clusters ruptured together with the failure of the H-bonds at active site (see Figure S2 (e) to (h)).

### ABT538

**Figure 5:**
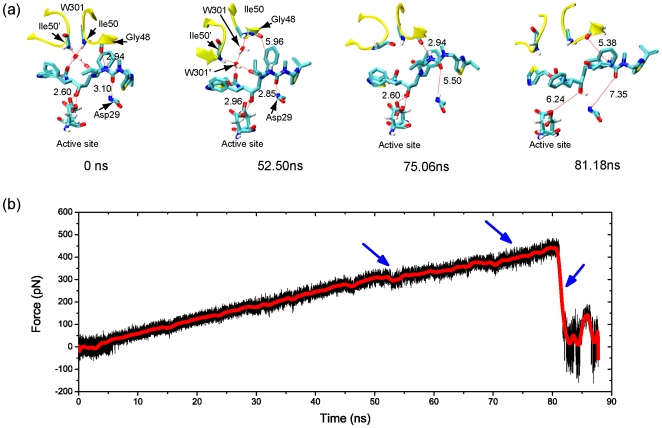
Dissociation process of ABT538 from the protease. (a) Snapshots of ABT538 escaping from the binding pocket of the protease under the external pulling forces. The numbers in the panel indicate the lengths of the H-bonds, and the simulation times are given at the bottom of each snapshot. (b) The pulling force of the ABT538 bound complex during the SMD simulation. The red curve is the average values with a running average time window of 500 ps. The blue arrows indicate the critical points of force dropping during the simulation.

**Figure 6:**
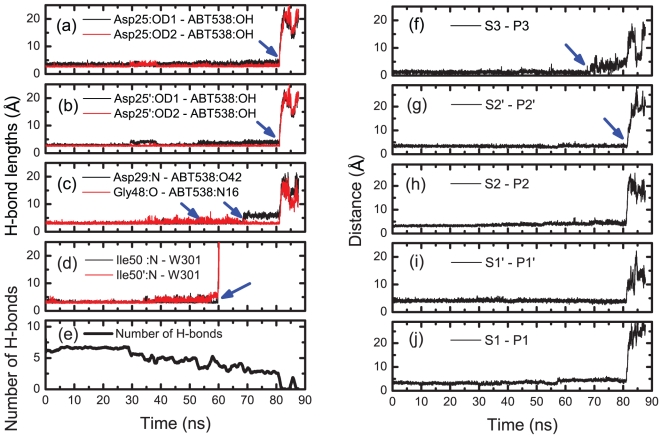
Analyses of the dynamics of H-bond interaction and hydrophobic interactions in ABT538 bound complex. (a) to (d): The H-bond lengths between the protease and the ABT538 during the pulling simulation. (e) Evolution of the number of H-bonds between the protease and the ABT538 during the simulation. (f) to (j): The distances between the subsites (S) of protease and the sidechains (P) of the inhibitor ABT538 during the simulation, which were used to monitor the state of the hydrophobic clusters. The blue arrows indicate the critical points of bond length change during the simulation.

In comparison with those in AHA001-PR and XK263-PR complexes, the failure process of the H-bond network in ABT538-PR complex was different. Figure 5 shows that the H-bond network was stable till the pulling force reaching a much higher value at *t*∼52.50 ns, while the H-bond number was about 6 (see Figure 6 (e)). Further increasing of the pulling force caused the rupture of the H-bonds between the flap tips and the water molecule W301, then W301 moved away from its original position together with one flap tip. Interestingly, another water molecule W301′ from the solvent got to the original position of W301, and re-built the H-bond connections between flap tips and ABT538 together with the original W301 molecule. The two water molecules W301 and W301′ formed a water chain between the flap tips and the ABT538, as shown in Figure 5 (a) at 52.50 ns (see also Figure 6 (d)). The water chain later ruptured under the pulling force at t∼60 ns, which cause only a slight drop of the pulling force, and the H-bond number became 4 (see Figure 6 (e)). Then, the H-bonds at lateral sides Asp29:N-ABT538:O42 and Gly48:O–ABT538:N16 came into play in keeping the stability of the H-bond network (see also Figure S3). With further increasing of the pulling force, the H-bond Asp29:N-ABT538:O42 ruptured at 75.06 ns, as shown in Figure 5 (a). However, the H-bond Gly48:O–ABT538:N16 was still stable. Therefore, the strength of interactions between the PR and inhibitor ABT538 are still strong enough to keep the stability of ABT538 in the binding pocket. At this stage, the H-bond number became 3 (see Figure 6 (e)), and the hydrophobic clusters were also stable. Finally, with further increase of the pulling force to a critical value, the H-bonds Asp25/Asp25′–ABT538 and Gly48:O–ABT538:N16, as well as the hydrophobic clusters between the subsites of PR and the sidechain of ABT538, ruptured together, and the inhibitor ABT538 was pulled out from the binding pocket (see Figures 5 and 6). We noted that the distances between the hydrophobic groups (e.g., S1-P1,S1′-P1′, S2-P2, S2′-P2′) increase abruptly to be as high as 20 Å at about 80 ns, together with the rupture of most of H-bonds.

### Rupture force

**Figure 7:**
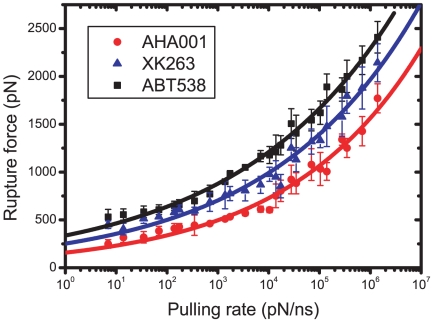
Rupture forces of the three inhibitor bound complexes calculated by the SMD simulations in term of pulling rates. The rupture force was defined as the highest peak loading force during the dissociation process in SMD simulations. The solid lines are the exponential fits according to pervious studies [45], [46] to guide the view.

The dissociation processes were simulated by systematically changing the pulling rate over six order of magnitude ranging form 1.4×10^6^ pN/ns to 6.95 pN/ns, focusing on the effect of the pulling rate on the rupture force. The rupture force was defined as the highest peak value of the pulling force as shown in Figure 3 and 5. Figure 7 shows that the rupture forces of the three inhibitor bound complexes change in exponential functions of the pulling rates, which can be fitted with the function 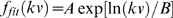 [45], consistent with pervious studies that the strength of molecular bonds increases as a weak power law of loading rate [46]. We note that the force level was very high because the pulling rates we used were much larger than those of experiments. If we choose the force rate as ∼100 pN/s which was usually used in experiments [19], [47], the rupture force can be predicted by this fitting function as ∼35.88 pN, 22.91 pN and 10.81 pN for ABT538, XK263 and AHA001, respectively. These results are in good agreement with experimental results of dissociation forces for antibody fragment-peptide complex (i.e. ∼35 pN) [19] and unfolding forces for coiled-coil myosin structure (i.e. ∼30 pN) [47] measured by AFM in similar force rate ranges. It can be seen that the curves of rupture force can quantitatively distinguish the binding strength of these three inhibitors. For example, the binding strength of ABT538 complex was stronger than that of XK263 complexes, and that of XK263 was stronger than that of AHA001 at different pulling rates. Our results were consistent with recent studies by Colizzi et al. [48] which indicated that stronger bound inhibitors yielded higher rupture forces, whereas the weaker inhibitors produced lower rupture forces.

The different binding strength or rupture forces among these three inhibitors can be understood by considering the failure mode of the H-bond network and the coordination between the H-bond network and the hydrophobic clusters during the dissociation process. As we can see, the cooperativity of H-bonds of ABT538 complex is more effective than those of AHA001/XK263 complexes. For example, in AHA001/XK263 complexes, one of the two H-bonds at flap tips ruptured before the H-bonds at active site, while the other one ruptured after those at active site. However, most of H-bonds in ABT538 bound complex rupture simultaneously. Our results were consistent with previous studies [49], [50], [51] which showed that the cooperativity of H-bonds facilitate to achieve strong binding strength in biomolecules. In addition, we showed that the earlier the failure of the H-bond network (especially the H-bonds at the active site), the lower the rupture force. For example, the failure of the H-bonds in AHA001 was earlier than that of XK263, and that of XK263 was earlier than that of ABT538. The underlying mechanism is that the coordination of H-bonds with the hydrophobic interactions between inhibitor and the PR is also crucial for the binding strength of inhibitors. If the role of the H-bond network was synchronized with that of the hydrophobic interactions, then the binding strength could be optimized, as in the case of the ABT538 complex. Because the H-bond is more sensitive to the displacement of inhibitor relative to the binding site, in order to improve the binding strength of inhibitors, the structure of the H-bond network should be optimized. In the ABT538 bound complex, we identified two mechanisms that can optimize the H-bond network. One is having more accessorial H-bonds at lateral sides of flap tips and active site for stabilizing the H-bond network, as shown in the section for ABT538. The other one is introducing water molecules W301/W301′. The water molecules can be helpful for the stability of the connection between the flap tips and the inhibitor with their flexible movement and rotation. These two mechanisms let the H-bond networks and hydrophobic interactions ruptured simultaneously in inhibitor ABT538 bound complex with higher binding strength than those in the AHA001 and XK263 bound complexes.

### Energy landscape

**Figure 8:**
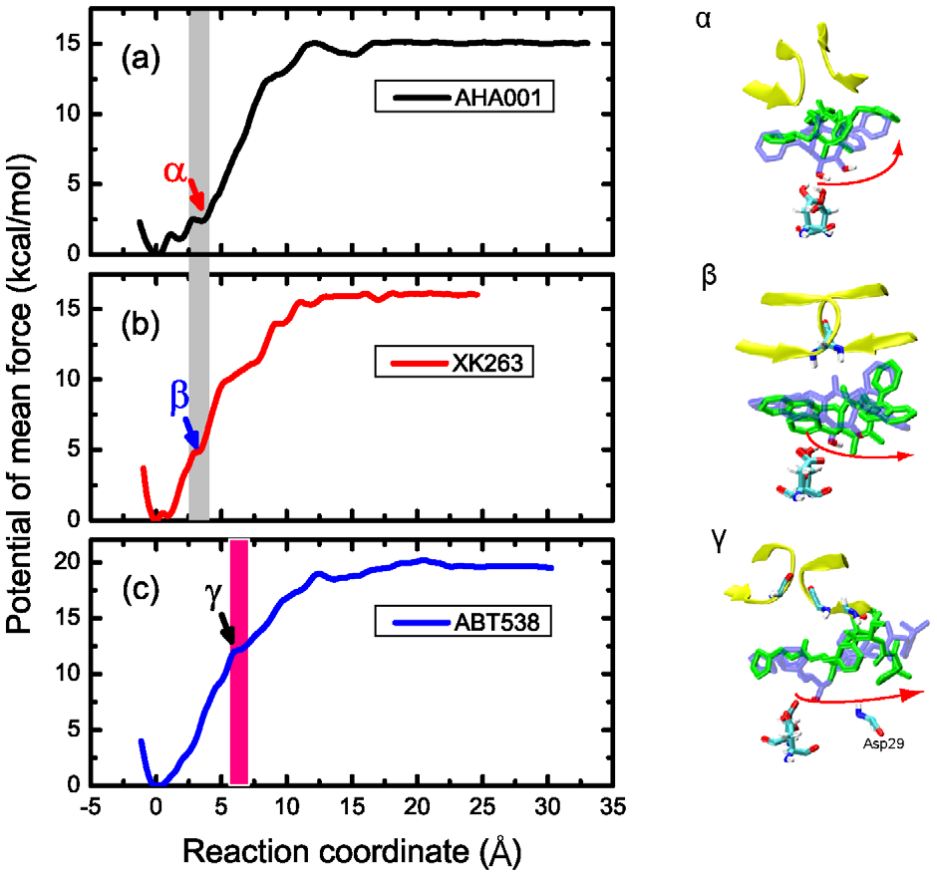
Energy landscape of dissociation of the three inhibitors from the binding pocket calculated with the umbrella sampling simulations. (a) AHA001; (b) XK263 and (c) ABT538. The reaction coordinate was along the pulling direction, of which the origin point corresponding to the tight structure that the inhibitor was at the right position of active site with intact H-bond networks and hydrophobic interactions. The color bands in (a), (b) and (c) indicate the positions that the H-bond networks ruptured in the complexes. The snapshots α, β and γ illustrate the conformational transitions of the complexes corresponding to the arrows pointed in (a) to (c). The inhibitors are represented by both blue and green rods to illustrate the movement tendencies.

The energy landscape of the dissociation process can be determined by using the umbrella sampling simulations, and the reaction coordinate corresponds to the pulling pathway (see Figure 1). By using approximate 40 sampling windows along this reaction coordinate, one-dimensional potential of mean force (PMF) curves were obtained for each system. Table 2 shows the parameters of the energy landscape of the three systems calculated from umbrella sampling method compared with corresponding experimental results. Figure 8 (a) clearly shows that there were several local minima in the energy landscape of AHA001 bound complex which were separated by a few *k_B_T* energy barriers. Therefore, the system states can transfer from one minimum to another, which was believed to be one of the main factors causing the instability of the system [48]. These local minima correspond to the drops in the pulling force (see Figure 3 (a)), which were caused by the instability of H-bond network. The position α in Figure 8 (a) corresponds to the failure of the H-bonds at the active site as well as the failure of the whole H-bond network (see also the snapshot α in Figure 8). Thereafter, the stability of the bound complex was maintained only by the hydrophobic clusters.

**Table 2:** The energy landscape profiles calculated by the umbrella sampling method in comparison with the experiments.^a^

Inhibitors	Umbrella sampling					Experiment values				
	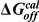 (kcal/mol)	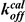 (s^−1^)	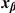 (Å)	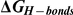 (kcal/mol)	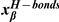 (Å)	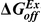 (kcal/mol)	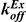 (s^−1^)	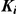 (nM)	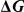 d (kcal/mol)	Refs.
AHA001	15.08	54.44	11.9	2.54	2.88	14.79	∼88.3^b^	12.2	−10.79	[3], [63]
						15.21	∼43.8^c^			
XK263	15.94	12.75	12.8	4.85	2.97	14.79	∼88.3^b^	0.31	−12.97	[3], [64]
						15.21	∼43.8^c^			
ABT538	20.20	9.59×10^−3^	12.6	12.12	6.23	21.08	2.16×10^−3^	0.59	−12.59	[3]

a As there is no experimental results for XK263 and AHA001 were determined, the kinetic behaviors of XK263 and AHA001 were represented by the data of their analog like inhibitors DMP323 and AHA008 (see Figure S4).

b Data from DMP323, *K_i_* = 0.27 nM.

c Data from AHA008, *K_i_* = 0.23 nM.

d The free energy 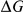 is obtained by 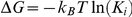.

For XK263 bound complex, there were also local minima in the energy landscape (see Figure 8 (b)). The position β in Figure 8 (b) corresponds to the failure of the H-bond network of XK263 bound complex (see also the snapshot β in Figure 8). The H-bond networks failed approximately at the same position along the reaction coordinate (about 3 Å) as that of the AHA001 bound complex.

Different from the cyclic urea inhibitors, there was only one energy well in the energy landscape of the ABT538 bound complex before the rupture of the H-bond network along the reaction coordinate. This suggested that the ABT538 bound complex was more stable than that of cyclic urea inhibitors (AHA001 and XK263) at the native position. Position γ in Figure 8 (c) shows the position in the energy landscape when the H-bond network ruptured (see also snapshot γ in Figure 8).

Here we define the width of the energy well of the H-bond network as the largest distances between the center of mass of inhibitor and the active site of PR above which there was no H-bond maintained in the complex. Figure 8 shows that the energy well width of the H-bond network of ABT538 complex is 6.23 Å that is over twice of those of the AHA001 and XK263 bound complexes, 2.88 Å and 2.97 Å, respectively (see Table 2 and positions α, β and γ in Figure 8 (a), (b) and (c)). In addition, the energy barrier for the rupture of the H-bond network of the ABT538 complex is much higher than those of the AHA001 and XK263 complexes (see Table 2). Therefore, the H-bond network was more stable in the ABT538 complex than those in the AHA001/XK263 complexes.

## Discussion

Using the SMD simulation method, we simulated the enforced dissociation processes of three different inhibitors, AHA001, XK263 and ABT538, from the binding pocket of the PR. We showed that the dissociation processes of these three inhibitors were different in several aspects including, e.g., the dissociation time, rupture force, and the failure modes of H-bonds and hydrophobic clusters as well as the coordination between these two interactions. The results showed that the enforced dissociation process of ABT538 was slower than those of the cyclic urea inhibitors AHA001 and XK263, and the rupture force of ABA538 was larger than those of the cyclic urea inhibitors.

We showed that these differences could be understood by studying the failure mechanisms of interaction between inhibitors and the PR at atomistic details. We suggested that the stability of H-bond network between inhibitors and the PR dominates the binding strength and therefore the dissociation rate of the inhibitors. Furthermore, the structure of the H-bond network, i.e., the distribution of H-bonds, dominates the stability of the H-bond network. We showed that the structure of the H-bond network of ABT538 bound complex is very different from that of the cyclic urea inhibitors. For example, besides the H-bonds at the flap tips and the active site, there are two additional H-bonds at the lateral sides of flap tips and the active site. In addition, there is a water molecule W301 which can help the rebinding of H-bonds at flap tips via its flexible movement. These special properties of the H-bond network in ABT538 complex make it possible to achieve the cooperativity of H-bonds in the H-bond network as well as the coordination between the H-bond network and the hydrophobic clusters, which ultimately determine the binding strength between inhibitors and the PR. To the best of our knowledge, this is the first study of dissecting the dissociation processes of inhibitor from the HIV-1 PR binding site by more quantitatively analyzing the stability of the H-bond network.

For further understanding the underlying physics of our findings, the experimental and numerical evidences supporting our results will be discussed as following. According to Table 2, we can see that the dissociation energy barrier 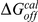 obtained by the umbrella sampling are in a good agreement with the values from experiments 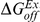 (see Figure S4 for molecular structure of relative inhibitors). The differences between the results of numerical calculations and experiments may come from the dissociation pathway we chosen. The natural dissociation pathway may be a somewhat tortuous one rather than the straight one we chose. Moreover, the dissociation rate constants 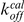 can be calculated from the dissociation free energy by applying the Arrhenius equation [52],
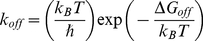
(1)where 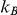 is the Boltzmann's constant, *T* is the absolute temperature and 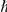 is the Planck's constant. The calculated dissociation rate constants 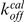 are in the same order of magnitude with the values from the experiments 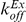. The results showed that the dissociation rate constant of ABT538 is much lower than those of the cyclic urea inhibitors AHA001 and XK263.

It was shown that the binding processes of inhibitors to HIV-1 PR exhibit a two-step process [53],
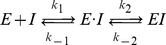
(2)where *E* represents the PR, *I* the inhibitor, and the group *E·I* and *EI* represent the loose and tight forms of the bound complexes, respectively. Note that 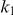, 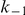 and 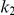, 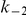 are the reaction rate constants of the first and second step, respectively. Previous MD studies [13], [54], [55] indicated that the second step, represented by 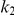 and 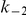, is mainly the conformational transitions and position adjustments of the protease and inhibitor in order to form a tight complex. In addition, pervious studies indicated that the dissociation rate constant 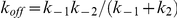, varying from 3×10^−5^ s^−1^ to 6×10^3^ s^−1^, is mainly dominated by the dissociation rate constant of the second step 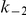 [56].

Our results are consistent with these studies. We showed that the hydrogen bond network in the AHA001/XK263 bound complexes ruptured faster than that in the ABT538 bound complex. Once the H-bond network ruptured, the bound complexes transfer from the tight form to the loose one, i.e., from *EI* to *E·I* as shown in Eq. (2). This demonstrated that the second step dissociation rate constants 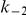 of the AHA001/XK263 bound complexes were larger than that of the ABT538 bound complex, therefore resulting in much faster total dissociation rate of AHA001/XK263 bound complexes than that of the ABT538 bound complex as shown by the experimental results [3]. To the best of our knowledge, our results for the first time explained the underlying mechanisms in atomistic details why the cyclic urea inhibitors often have fast dissociation rates shown by experiments [3].

It is noteworthy that the theoretical models developed by Dudko et al. [25], [26] and Hummer & Szabo [57], as well as the models by Bell [58] and Evans and Ritchie [46] can also be used to calculate the energy barrier. Compared to these theoretical models, the umbrella sampling method not only can calculate the energy barrier, but also can directly calculate the profile of the energy landscape. For the details of the comparison between these theoretical models and the umbrella sampling method see Table S2. In addition, the Jarzynski's equality method can also be used to calculate the shape of one-dimensional free energy landscape [30], [59], but it needs a large number of pulling trajectories in order to reproduce the shape of free energy landscape accurately [60]. To obtain a reliable energy landscape it may require an order of magnitude longer computing time compared to the umbrella sampling method [60]. For the details of the comparison between the Jarzynski's equality method and the umbrella sampling method see Figure S5.

**Figure 9:**
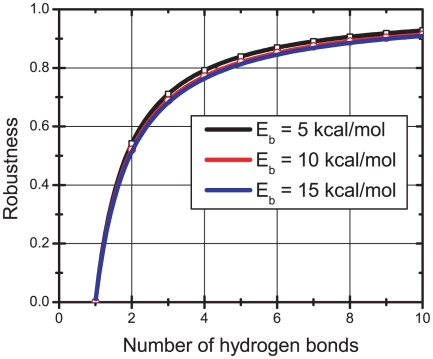
Robustness of the H-bond networks in term of the H-bond number. The three curves in the panel show that there are no significant differences in the robustness value when the single bond energy barriers (effective) change between 5∼15 kcal/mol.

To further quantitatively describe the stability of the H-bond network, we here introduce the concept of the robustness. The robustness of the H-bond network was defined as the ratio of strength of the network with one H-bond broken to that of the intact network, which is given by [61]

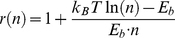
(3)where 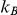 is the Boltzmann constant, *T* is the absolute temperature, 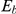 is the energy barrier of a single H-bond and *n* is the number of H-bonds considered in the system. The 0% robustness means that the bond network is highly fragile, and the 100% robustness indicates the bond network is highly robust with maximum fault tolerance. In the cyclic urea inhibitors (AHA001/XK263) complex, there are about 4 H-bonds in the H-bond network (see Table 1). As a result, the robustness of the cyclic urea inhibitor bound complex H-bond network is about 79% (see Figure 9). In comparison, there are about 6 H-bonds in the H-bond network of ABT538 bound complex. And the robustness of the ABT538 bound complex H-bond network is about 87% (see Figure 9), which is higher than that of cyclic urea inhibitor bound ones. Thus, the stability of the H-bond network would be less influenced if one H-bond ruptured in ABT538 bound complex in comparison with the cyclic urea inhibitor bound complexes. High robustness will help increase the reforming probability of ruptured H-bonds. Therefore, the H-bond network in ABT538 bound complex represents larger fault tolerance capability.

The robustness is crucial for the potent inhibitor design by considering the frequent mutation of the virus. Because the H-bond strength was highly sensitive to the bond length (distance between the donor and receptor) [62], mutation can significantly influence the stability of the H-bond network. Previous studies [15] showed that the mutations of the protease can decrease the effectiveness of the inhibitors by substantially increasing the dissociation rate constants (*k_off_*). Therefore, the H-bond network with high robustness is highly desirable for the design of potent inhibitors to resist the effects of mutation. The high robustness will also allow the coordination between the H-bond networks and the hydrophobic interactions, which can further enhance the stability of the H-bond networks.

In summary, we studied the entire dissociation processes of inhibitors from HIV-1 PR using the SMD simulations and umbrella sampling simulations with explicit water model. The stability of H-bond network was analyzed quantitatively to understand the underlying mechanisms of the significant differences in dissociation rate constants among different inhibitors. We showed that the binding strengths of different inhibitors, e.g., AHA001, XK263 and ABT538, can be distinguished by the rupture forces quantitatively from the SMD simulations. Detailed analysis of the dissociation processes of inhibitors from the PR binding pocket showed that the different binding strength was caused by the difference in the stability of H-bond networks in the bound complexes. Compared with the cyclic urea inhibitors complexes, there are more H-bonds at the lateral sides of the flaps and active sites in the ABT538 bound complex. In addition, the water molecule W301 enhances the rebinding of the hydrogen bonds at the flap tips through its flexible movement. Because of these two superior structural features, the H-bond network in ABT538 bound complex shows higher robustness and stability than those of the cyclic urea inhibitor complexes. The high stability of the H-bond network allows it to have a harmonic coordination with the hydrophobic cluster so that they can work together to resist the dissociation. This study presents a microscopic picture at atomistic details for explaining the large difference in the dissociation rate constant among different inhibitors, which might provide important guidelines for design of the novel potent inhibitors with optimized interactions.

## Supporting Information

Figure S1 **Dissociation process of XK263 from the protease.** (a) Snapshots of XK263 escaping from the binding pocket of the protease under the external pulling forces. The numbers in the panel indicate the lengths of the hydrogen bonds, and the simulation times were given at the bottom of each snapshot. (b) The pulling force of XK263 bound complex during the SMD simulation. The red curve is the average values with a running average time window of 500 ps. The blue arrows indicate the critical points of force dropping during the simulation.
(TIF)

Figure S2 **Analyses of the dynamics of H-bond interaction and hydrophobic interactions in XK263 bound complex.** (a) to (c) The H-bond lengths between the protease and the inhibitor XK263 during the pulling simulation. (d) Evolution of the number of H-bonds between the protease and the inhibitor XK263 during the simulation. (e) to (h) The distances between the subsites (S) of protease and the sidechains (P) of XK263 during the simulation, which were used to monitor the state of the hydrophobic clusters. The blue arrows indicate the critical points of bond length change during the simulation.
(TIF)

Figure S3 **(a) Pulling force of ABT538 bound complex with different pulling rates.** The pulling distance was defined by the displacement of the “dummy” atom relative to its original position during the SMD simulations. The arrows indicate the time of the hydrogen bonds rupture between the inhibitor ABT538 and residue Asp29, Gly48. (b) Snapshots of the rupture of the hydrogen bond formed by ABT538 and residue Asp29.
(TIF)

Figure S4 **Molecular structures of two cyclic urea inhibitors. (a) DMP323 and (b) AHA008.**
(TIF)

Figure S5
**(a) Free energy landscape of inhibitor ABT538 bound complex obtained by using Jarzynski's equality and umbrella sampling methods.** The result by Jarzynski's equality was obtained from 100 pulling trajectories with 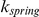 = 694.8 pN/nm and pulling velocity 0.5 nm/ns. The parameter *n* is the number of different work values chosen at random from the total 100 trajectories, using the block averaging method. The value of n→∞ is the linear extrapolation based on the 100 pulling trajectories. (b) The work distribution in the 100 pulling trajectories at reaction coordination 20 Å.(EPS)

Table S1 Force constants and pulling velocities used in the SMD simulations.
(DOC)

Table S2 The energy barrier of AHA001 bound complex predicted by different models/methods.
(DOC)
