# Supplementary figures and images for: Strength of Hydrogen Bond Network Takes Crucial Roles in the Dissociation Process of Inhibitors from the HIV-1 Protease Binding Pocket
Source: PLoS One. 2011 Apr 29;6(4):e19268. doi: 10.1371/journal.pone.0019268 (PMC3084818; doi:10.1371/journal.pone.0019268)

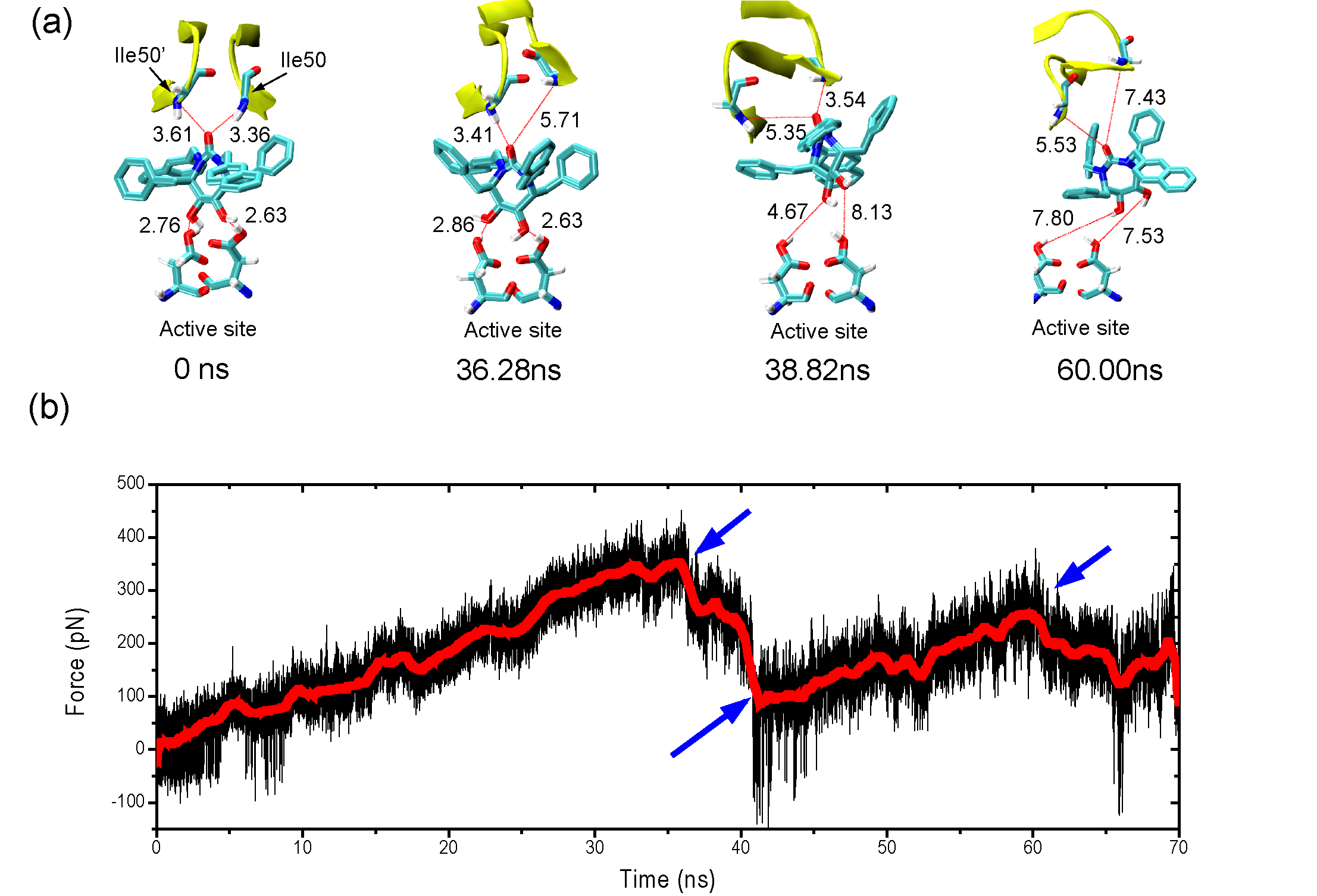

Supplement: Figure S1 — Dissociation process of XK263 from the protease. (a) Snapshots of XK263 escaping from the binding pocket of the protease under the external pulling forces. The numbers in the panel indicate the lengths of the hydrogen bonds, and the simulation times were given at the bottom of each snapshot. (b) The pulling force of XK263 bound complex during the SMD simulation. The red curve is the average values with a running average time window of 500 ps. The blue arrows indicate the critical points of force dropping during the simulation. (TIF) [file pone.0019268.s001.tif]

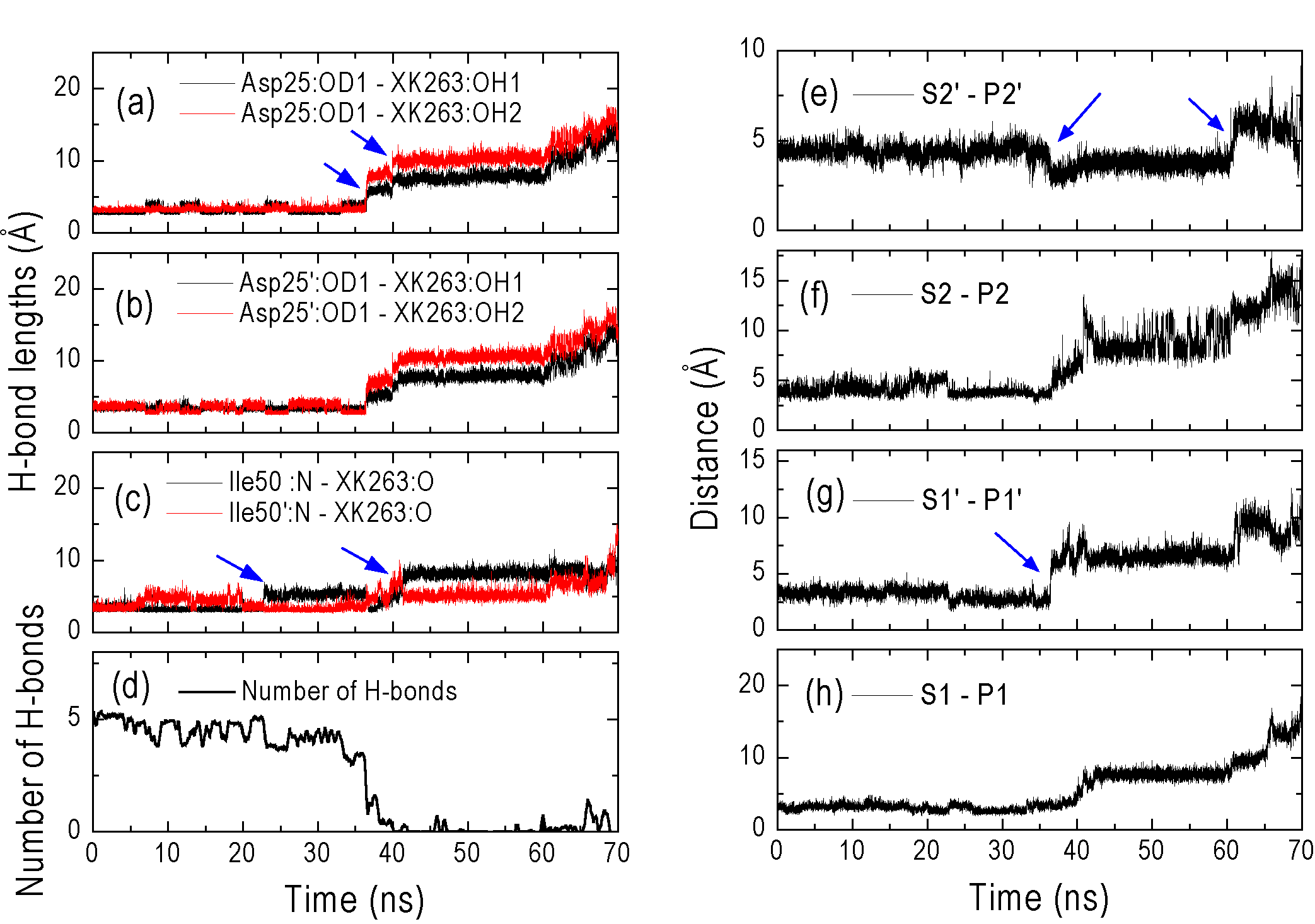

Supplement: Figure S2 — Analyses of the dynamics of H-bond interaction and hydrophobic interactions in XK263 bound complex. (a) to (c) The H-bond lengths between the protease and the inhibitor XK263 during the pulling simulation. (d) Evolution of the number of H-bonds between the protease and the inhibitor XK263 during the simulation. (e) to (h) The distances between the subsites (S) of protease and the sidechains (P) of XK263 during the simulation, which were used to monitor the state of the hydrophobic clusters. The blue arrows indicate the critical points of bond length change during the simulation. (TIF) [file pone.0019268.s002.tif]

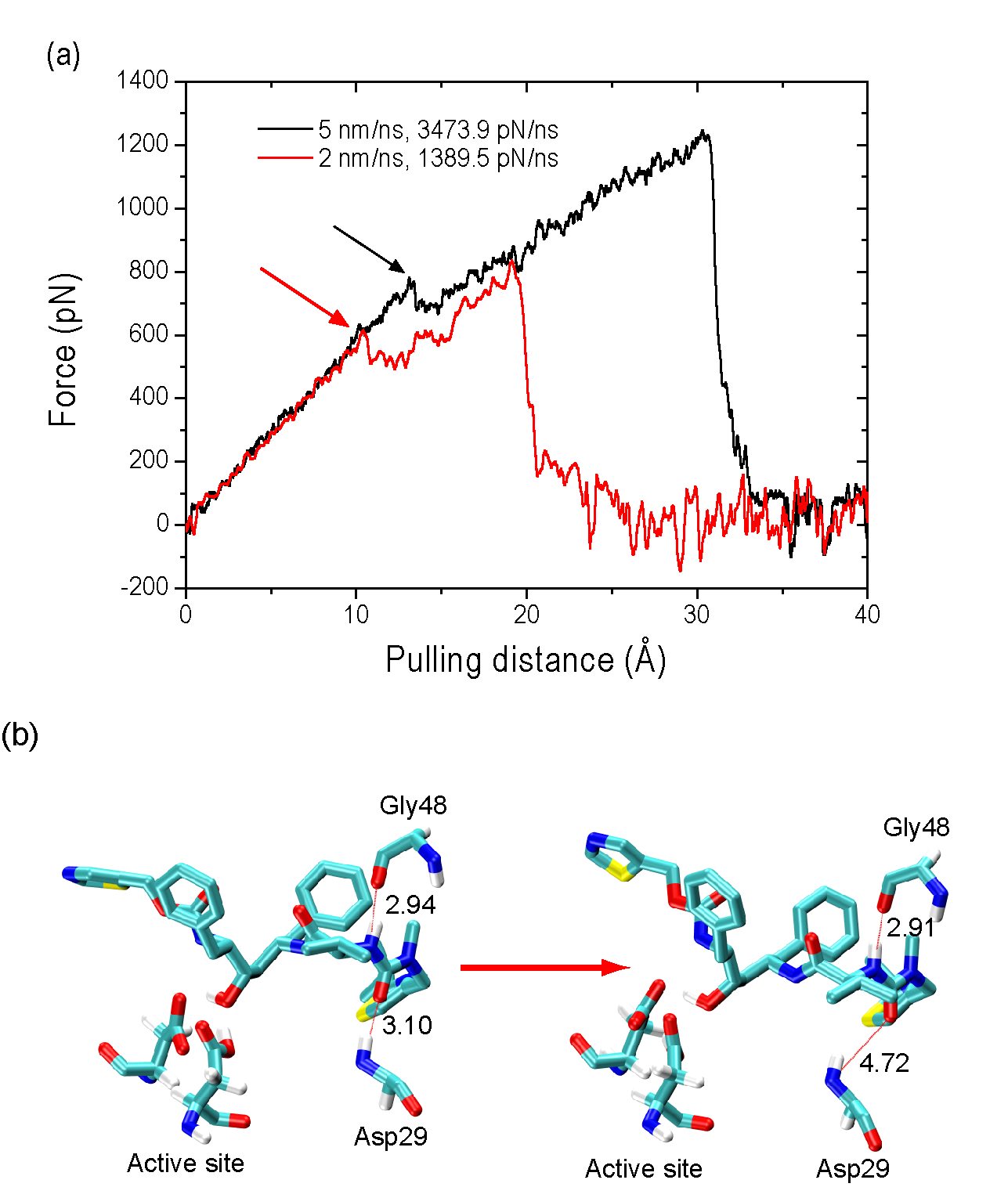

Supplement: Figure S3 — (a) Pulling force of ABT538 bound complex with different pulling rates. The pulling distance was defined by the displacement of the “dummy” atom relative to its original position during the SMD simulations. The arrows indicate the time of the hydrogen bonds rupture between the inhibitor ABT538 and residue Asp29, Gly48. (b) Snapshots of the rupture of the hydrogen bond formed by ABT538 and residue Asp29. (TIF) [file pone.0019268.s003.tif]
