# Supplementary material for: Strength of Hydrogen Bond Network Takes Crucial Roles in the Dissociation Process of Inhibitors from the HIV-1 Protease Binding Pocket
Source: PLoS One. 2011 Apr 29;6(4):e19268. doi: 10.1371/journal.pone.0019268 (PMC3084818; doi:10.1371/journal.pone.0019268)
Supplement: Table S1 — Force constants and pulling velocities used in the SMD simulations. (DOC) [file pone.0019268.s006.doc]

Table S1. Force constants and pulling velocities used in the SMD simulations.

| Spring constant  (pN/nm) | Pulling velocity  (nm/ns) | Force loading rate  (pN/ns) | Simulation times |
| --- | --- | --- | --- |
| 6947.7 | 200 | 1.39×10^6^ | 10 |
|  | 100 | 6.95×10^5^ | 10 |
|  | 40 | 2.78×10^5^ | 10 |
|  | 20 | 1.39×10^5^ | 10 |
|  | 15 | 1.04×10^5^ | 10 |
|  | 10 | 6.95×10^4^ | 5 |
|  | 5 | 3.47×10^4^ | 5 |
|  | 2 | 1.39×10^4^ | 5 |
|  | 1.5 | 1.04×10^4^ | 5 |
|  | 1 | 6.95×10^3^ | 5 |
|  | 0.5 | 3.47×10^3^ | 2 |
|  | 0.2 | 1.39×10^3^ | 2 |
|  | 0.1 | 6.95×10^2^ | 2 |
|  | 0.05 | 3.47×10^2^ | 2 |
|  | 0.02 | 1.39×10^2^ | 2 |
| 3473.9 | 200 | 6.95×10^5^ | 10 |
|  | 100 | 3.47×10^5^ | 10 |
|  | 40 | 1.39×10^5^ | 10 |
|  | 20 | 6.95×10^4^ | 10 |
|  | 10 | 3.47×10^4^ | 5 |
|  | 5 | 1.74×10^4^ | 5 |
|  | 2 | 6.95×10^3^ | 5 |
|  | 1 | 3.47×10^3^ | 5 |
|  | 0.5 | 1.74×10^3^ | 2 |
|  | 0.2 | 6.95×10^2^ | 2 |
|  | 0.1 | 3.47×10^2^ | 2 |
|  | 0.05 | 1.74×10^2^ | 2 |
|  | 0.02 | 6.95×10^1^ | 2 |

Table S1 continued.

| Spring constant  (pN/nm) | Pulling velocity  (nm/ns) | Force loading rate  (pN/ns) | Simulation times |
| --- | --- | --- | --- |
| 694.8 | 200 | 1.39×10^5^ | 10 |
|  | 100 | 6.95×10^4^ | 10 |
|  | 40 | 2.78×10^4^ | 10 |
|  | 20 | 1.39×10^4^ | 10 |
|  | 15 | 1.04×10^4^ | 10 |
|  | 10 | 6.95×10^3^ | 5 |
|  | 5 | 3.47×10^3^ | 5 |
|  | 2 | 1.39×10^3^ | 5 |
|  | 1 | 6.95×10^2^ | 5 |
|  | 0.5 | 3.47×10^2^ | 2 |
|  | 0.2 | 1.39×10^2^ | 2 |
|  | 0.1 | 6.95×10^1^ | 2 |
|  | 0.05 | 3.47×10^1^ | 2 |
|  | 0.02 | 1.39×10^1^ | 2 |
| 347.4 | 200 | 6.95×10^4^ | 10 |
|  | 100 | 3.47×10^4^ | 10 |
|  | 40 | 1.39×10^4^ | 10 |
|  | 20 | 6.95×10^3^ | 10 |
|  | 10 | 3.47×10^3^ | 5 |
|  | 5 | 1.74×10^3^ | 5 |
|  | 2 | 6.95×10^2^ | 5 |
|  | 1 | 3.47×10^2^ | 5 |
|  | 0.5 | 1.74×10^2^ | 2 |
|  | 0.2 | 6.95×10^1^ | 2 |
|  | 0.1 | 3.47×10^1^ | 2 |
|  | 0.02 | 6.95 | 2 |
