# Supplementary material for: Strength of Hydrogen Bond Network Takes Crucial Roles in the Dissociation Process of Inhibitors from the HIV-1 Protease Binding Pocket
Source: PLoS One. 2011 Apr 29;6(4):e19268. doi: 10.1371/journal.pone.0019268 (PMC3084818; doi:10.1371/journal.pone.0019268)
Supplement: Table S2 — The energy barrier of AHA001 bound complex predicted by different models/methods. (DOC) [file pone.0019268.s007.doc]

Table S2. The energy barrier of AHA001 bound complex predicted by different models/methods.

|  | $\left\langle F\right\rangle\propto\left(\ln KV\right)^{\mu}$ | | | Umbrella sampling | Experiment |
| --- | --- | --- | --- | --- | --- |
|  | $\mu=\frac{1}{2}$ | $\mu=\frac{2}{3}$ | $\mu=1$ |  |  |
| *E_b_* (kcal/mol) | 10.47 | 7.99 | 9.50 | 15.08 | 14.79 |
| *k_off_* (s^-1^) | 8.34×10^3^ | 3.36×10^5^ | 7.46×10^5^ | 54.44 | 88.3 |
| *x_β_* (Å) | 1.79 | 1.08 | 0.84 | 11.9 | -- |
